# Management of mung bean leaf spot disease caused by *Phoma herbarum* through *Penicillium janczewskii* metabolites mediated by MAPK signaling cascade

**DOI:** 10.1038/s41598-023-30709-6

**Published:** 2023-03-03

**Authors:** Shazia Shafique, Ume Attia, Sobiya Shafique, Bushra Tabassum, Naureen Akhtar, Ayman Naeem, Qamar Abbas

**Affiliations:** 1 Department of Plant Pathology, Faculty of Agricultural Sciences, University of the Punjab, Lahore, 54590 Pakistan; 2 School of Biological Sciences, Faculty of Life Sciences, University of the Punjab, Lahore, 54590 Pakistan; 3 Monash University, Melbourne, Australia

**Keywords:** Biological techniques, Molecular biology, Physiology

## Abstract

*Vigna radiata* L., an imperative legume crop of Pakistan, faces hordes of damage due to fungi; infecting host tissues by the appressorium. The use of natural compounds is an innovative concern to manage mung-bean fungal diseases. The bioactive secondary metabolites of *Penicillium* species are well documented for their strong fungi-static ability against many pathogens. Presently, one-month-old aqueous culture filtrates of *Penicillium janczewskii, P. digitatum, P. verrucosum, P. crustosum,* and *P. oxalicum* were evaluated to check the antagonistic effect of different dilutions (0, 10, 20, … and 60%). There was a significant reduction of around 7–38%, 46–57%, 46–58%, 27–68%, and 21–51% in *Phoma herbarum* dry biomass production due to *P. janczewskii, P. digitatum, P. verrucosum, P. crustosum,* and *P. oxalicum,* respectively. Inhibition constants determined by a regression equation demonstrated the most significant inhibition by *P. janczewskii.* Finally, using real-time reverse transcription PCR (qPCR) the effect of *P. Janczewskii* metabolites was determined on the transcript level of *StSTE12* gene involved in the development and penetration of appressorium. The expression pattern of the *StSTE12* gene was determined by percent Knockdown (%KD) expression that was found to be decreased i.e. 51.47, 43.22, 40.67, 38.01, 35.97, and 33.41% for *P. herbarum* with an increase in metabolites concentrations viz., 10, 20, 30, 40, 50 and 60% metabolites, respectively. In silico studies were conducted to analyze the role of Ste12 a transcriptional factor in the MAPK signaling pathway. The present study concludes a strong fungicidal potential of Penicillium species against *P. herbarum.* Further studies to isolate the effective fungicidal constituents of Penicillium species through GCMS analysis and determination of their role in signaling pathways are requisite.

## Introduction

Mung bean (*Vigna radiata* L.) is a standout amongst the most imperative and critical pulse crops of Pakistan. It belongs to the family Fabaceae^1^ and developed from the tropical to sub-tropical territories in the world^2,3^. There are in excess of about five hundred varieties of pulses that assume a helpful part in increasing the fertility of the soil by a relationship with nitrogen-fixing bacteria. Seeds of pulses are profitable nutritional sources and are thought to be contrasting options to meat as they contain proteins (20 to 30% of dry weight). Seeds additionally have a low-fat substance (about 5%), fibers, sugars, calcium, zinc, and folic acid^4–6^. The mung bean seeds contain 1.30% fats, 24.20% protein contents, 60.4% starches; phosphorus (P) 340 mg, and calcium (Ca) is 118 mg for each 100 g of seed^7^. Besides, in mung bean seeds the protein content is two times higher than in the seeds of maize, with the least storage protein content (7 to 10%)^8,9^. Mung bean is a significant measure of bioactive Phyto synthetic substances. With expanding clinical confirmation proposing that mung bean plants have different potential advantages for health, their utilization has been developing at a rate of 5 to 10% every year^10^. It is notable for its detoxification exercises and is utilized to invigorate mindset, mitigate warm stroke, and diminish swelling in the late spring. Mung bean was recorded to be valuable in the direction of gastrointestinal disturbance and skin motorization^11^. Additionally, the seeds and sprouts of mung beans are generally utilized as a new serving of mixed green vegetables or as regular sustenance food in Pakistan, India, Bangladesh, South East Asia, and western nations^12^. Mung bean is developed in the biggest pulse region in Pakistan just second to chickpea^13^. Pakistan imports a very high number of legumes to cover the breach in demand and supply of pulses.

Plant diseases reduce the yield and productivity of several crops all over the world including mung bean. Yield losses because of the absence of plant security measures change from 46 to 96% contingent upon any crop varieties. Biotic diseases harm plants in different life forms, viz., insects, weeds, nematodes, allelopathic chemicals, and so on. Among these, fungi and viruses are the biggest and the most critical gatherings influencing all parts of the plant at all phases of the development of the food legumes^14^. Fungi are the most harmful pathogens to mung beans and cause diverse infections like leaf spots (*Cercospora* leaf spots and *Alternaria* leaf spot etc.), *Phytophthora* stem blight, powdery mildew, and wilting^15–17^ etc.

Most plant pathogenic fungi have the capability to rupture the primary cuticles of host plants by developing appressoria which can be either single-celled assemblies or multiple appressoria, that mutually form structures recognized as infection cushions^18,19^. The appressorium is a specialized infection structure that is crucial for penetration into the host cell^20^. Appressorium development is a complicated procedure comprising various signals, including physical and chemical stimuli. The mitogen-activated protein kinase (MAPK) cascade is responsible for the morphogenesis, conidiation, appressorium establishment, and pathogenicity of numerous fungi, including *Magnaporthe grisea*^21^, *Pyrenophora teres*^22^, *Colletotrichum* spp.^23^, *Botrytis cinerea*^24^, and so on. Generally, three types of MAPK signaling cascades exist in the filamentous fungi which include; (i) Slt2-homolog, (ii) Hog1-homolog, and, (iii) Fus3/Kss1-homolog MAPK. The latter one is essential for pathogenicity and virulence^25^. In the budding yeast *Saccharomyces cerevisiae*, five MAPK pathways are known to regulate mating, invasive growth, cell wall integrity, hyper osmoregulation, and ascospore formation^26^. In the plant fungal pathogen model *Magnaporthe oryzae* (previously known as *M. grisea*), appressorium establishment is mediated by hydrophobic surface induction^27^. Ste12 is a homeodomain transcription factor and, is a key target of MAPK signaling pathway during invasive growth in the filamentous fungi^28^. Moreover, Ste12 is regulated by kinases included Fus3/Kss1 in the MAPK signaling cascade that regulate activation or repression of the mating pathways in filamentous fungi in response to pheromone and starvation^29^. Ste12 homologs in filamentous plant pathogens mainly regulate penetration, intrusive growth, and disease formation. On the other hand, different fungal pathogens are varied in their pathogenic mechanisms^24^. In true filamentous fungi, Ste12-like proteins play essential roles in sexual development and pathogenicity. Interestingly, Ste12 and Ste12-like factors are important for pathogenesis in all animal and plant pathogens tested so far, and further functional analyses revealed their importance in the setting up of a pathogenicity genetic program specific to the host. This indicates that Ste12 genes are required for these developmental processes, which accompany the invasive colonization of a new environment^30^.

Biological control is used as a technique for controlling fungal diseases as being environmentally friendly and non-lethal to the health of human beings, livestock, and wildlife; particularly now that the entire world is screaming for IPM methods of pest control. A number of researchers reported that many plants and microorganisms contain antifungal compounds^31–33^. Substances that are extracted from different parts of plants i.e. root, stem, leaves, bark, flower, fruit and seed, and essential oils (terpenes,) and by the microorganisms i.e. bacteria, fungi, etc. have antimicrobial properties^32–38^. Some species of fungi secrete secondary metabolites which possess the very specified activity and can be toxic to specific groups or groups of organisms. Fungi are known to have great potential as a biocontrol agent against pests since 1963^39,40^. These days, fungal biological control is thought to be a quickly developing characteristic phenomenon in modern research for better plant yield^41^. Penicillium is a predictable source of bioactive metabolites. Penicillium species secrete an expanded range of extracellular active secondary metabolites, having effective mycotoxins as well as antibacterial and antifungal properties^42,43^. A number of Penicillium species have been reported to have antagonistic potential against many fungal pathogens hence, are used to control fungal diseases like root rot of Okra caused by *Fusarium solani*^44^, charcoal rot of Sorghum caused by *Macrophomina phaseolina*^45^, Cercospora leaf spot of Sugar beet caused by *Cercospora beticola*^46^, rice blast caused by *Pyricularia oryzae*^47^, charcoal rot of Mung bean caused by *Macrophomina phaseolina*^48^, etc. Few Penicillium species are well recognized because of their antagonistic activity against pathogens by producing antibiotics and persuading resistance in their hosts by triggering various defense signals^49^.

Keeping in view the problems of pathogens; the main objective of the present study was to evaluate the efficacy of extracellular secondary metabolites from some *Penicillium* species for their eventual use.

## Results

### Effect of metabolites of *Penicillium* species on fungal biomass production

Penicillium species metabolites significantly reduced the biomass production of the target pathogen. However, variability in the effect of metabolite extracts was observed.

#### Effect of metabolites of *Penicillium janczewskii*

**Figure 1:**
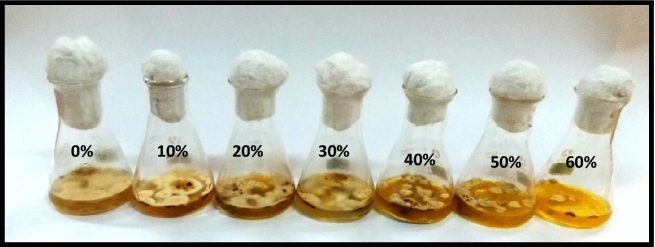
Effect of metabolite concentrations of *P. janczewskii* on the growth of *P. herbarum.*

**Figure 2:**
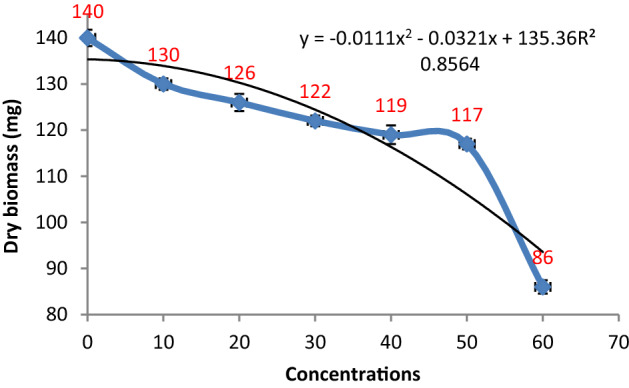
Effect of different metabolite concentrations of *P. janczewskii* on the biomass production of *P. herbarum.* Vertical bars show standard errors of the means of three replicates.

The antifungal potential of *P. janczewskii* was evaluated against *P. herbarum* where the obtained data revealed a sharp reduction in fungal growth production with the increase in the concentration of *P. janczewskii* extract. The fungal growth and the extract concentrations demonstrated a nonlinear relationship with R^2^ = 0.8564. Overall, a 7–38% reduction in fungal biomass production was observed over the control (Figs. 1 and 2)*.*

#### Effect of metabolites of *P. digitatum*

**Figure 3:**
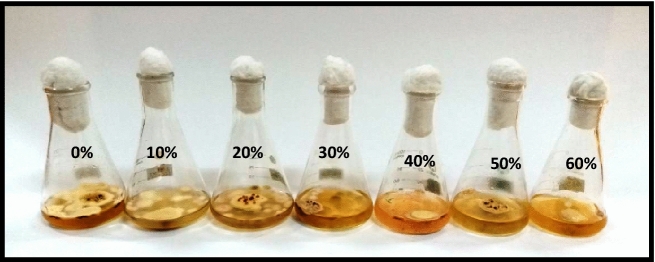
Effect of metabolite concentrations of *P. digitatum* on the growth of *P. herbarum.*

**Figure 4:**
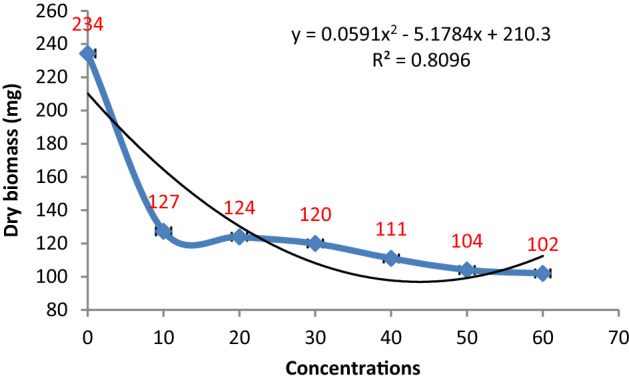
Effect of different metabolite concentrations of *P. digitatum* on the biomass production of *P. herbarum.* Vertical bars show standard errors of the means of three replicates.

Antifungal activity of various concentrations of *P. digitatum* on the biomass production of *P. herbarum* was evident from the results obtained as all the concentrations significantly retarded the growth of the targeted pathogen gradually. A non-linear relationship was recorded between fungal biomass and extract concentrations with R^2^ = 0.8096. The lowest concentration (10%) of *P. digitatum* extract proved very effective as it induced approximately 46% suppression in fungal biomass production while the highest concentration of 60% demonstrated about 57% biomass inhibition (Figs. 3 and 4).

#### Effect of metabolites of *P. verrucosum*

**Figure 5:**
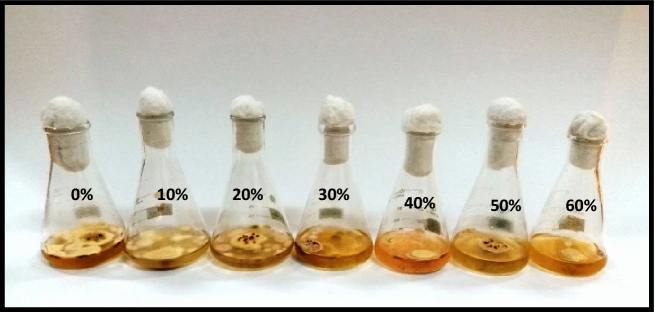
Effect of metabolite concentrations of *P. verrucosum* on the growth of *P. herbarum.*

**Figure 6:**
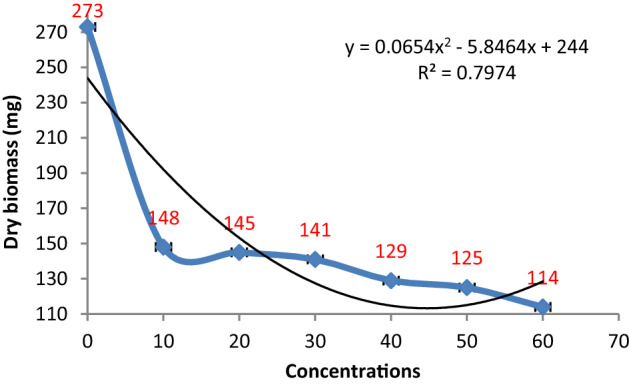
Effect of different metabolite concentrations of *P. verrucosum* on the biomass production of *P. herbarum.* Vertical bars show standard errors of the means of three replicates.

The results obtained from the biomass assays of *P. herbarum* in different metabolites concentrations of *P. verrucosum* exhibited a similar pattern of growth inhibition as depicted by *P. digitatum* (Fig. 5). The fungal biomass showed a nonlinear relationship between biomass and extract concentrations with R^2^ = 0.7974. The lowest concentration i.e. 10% of *P. verrucosum* extract exhibited a sharp decline of approximately 47% in fungal biomass production. The higher concentrations (20–50%) resulted in growth inhibition in the range of 47–55% with some insignificant differences. While the maximum arrest of about 58% in fungal biomass production was evidenced at the highest concentration (60%) of the employed extract (Fig. 6).

#### Effect of metabolites of *P. crustosum*

**Figure 7:**
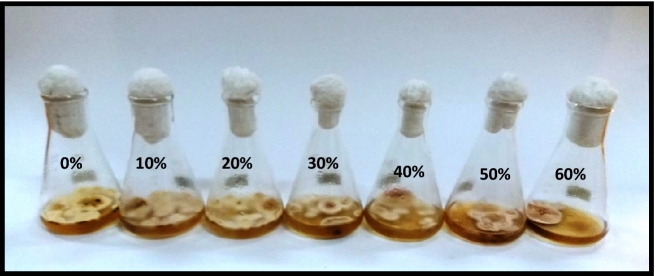
Effect of metabolite concentrations of *P. crustosum* on the growth of *P. herbarum.*

**Figure 8:**
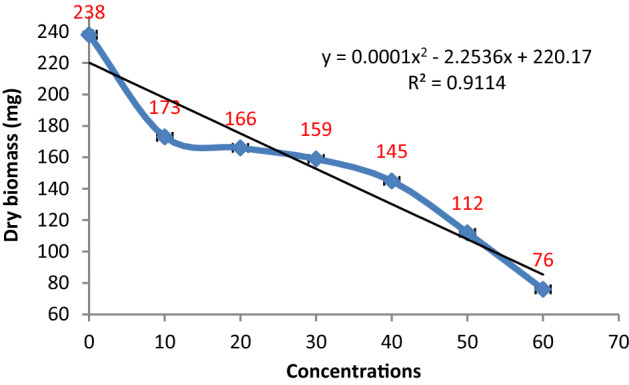
Effect of different metabolite concentrations of *P. crustosum* on the biomass production of *P. herbarum*. Vertical bars show standard errors of the means of three replicates.

Data pertaining to the effect of different concentrations of *P. crustosum* on the biomass production of *P. herbarum* depicted that the growth of the targeted pathogen was found to be retarded gradually with the increase in metabolites concentrations. The relationship between fungal biomass and the employed extract concentration is nonlinear with R^2^ = 0.9114. The lower concentrations i.e. 10–30% demonstrated the suppression in biomass production in the range of 27–33% while the higher concentrations of 40–60% caused approximately 40–68% reduction in fungal biomass production (Figs. 7 and 8).

#### Effect of metabolites of *P. oxalicum*

**Figure 9:**
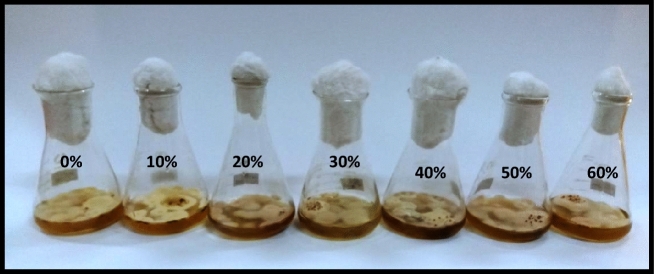
Effect of metabolite concentrations of *P. oxalicum* on the growth of *P. herbarum.*

**Figure 10:**
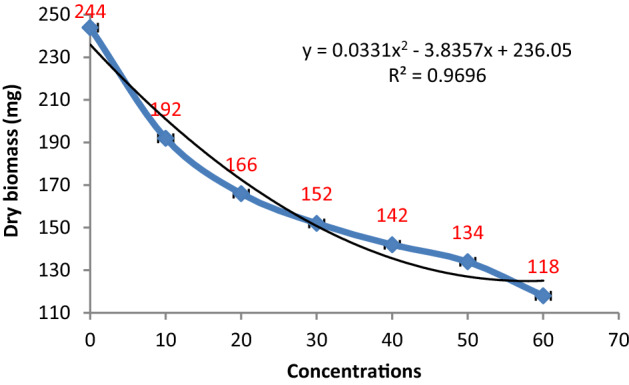
Effect of different metabolite concentrations of *P. oxalicum* on the biomass production of *P. herbarum.* Vertical bars show standard errors of the means of three replicates.

Metabolite extract obtained from *P. oxalicum* reduced the growth of *P. herbarum* in all concentrations in an almost similar manner as depicted by other species of Penicillium. A nonlinear relationship was displayed between the biomass of the target fungus and extract concentration with R^2^ = 0.9696. The lowest concentration (10%) induced aba out 21% decline in fungal growth production. The effect of 20–30% concentrations of *P. oxalicum* was significantly higher than this i.e. in the range of 32–38%. However, 40 and 50% concentrations showed an insignificant reduction in biomass production among each other but significant with respect to control and the lower concentrations treatments. Conversely, the maximum reduction of about 51% was observed at the highest concentration i.e. 60% (Figs. 9 and 10).

### Determination of kinetic constants of inhibition for *P. herbarum*

**Figure 11:**
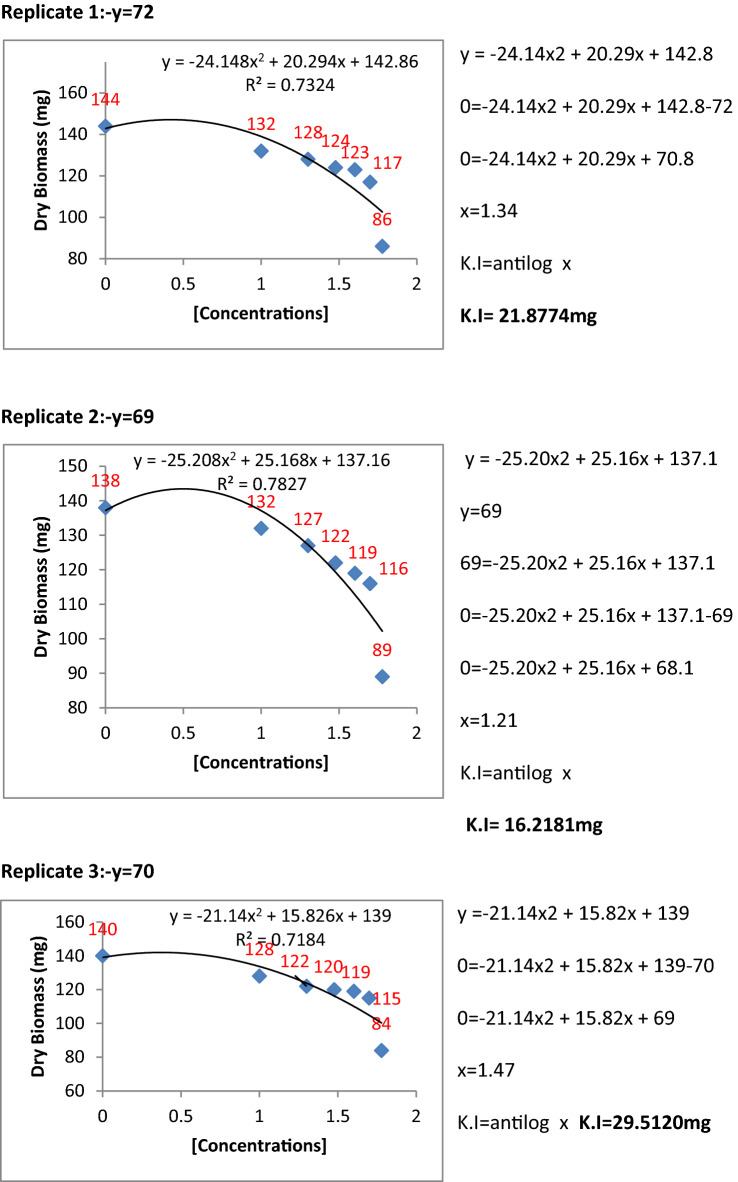
Kinetic constants of inhibition of *P. herbarum* by metabolites of *P. janczewskii* (3 replicates).

**Figure 12:**
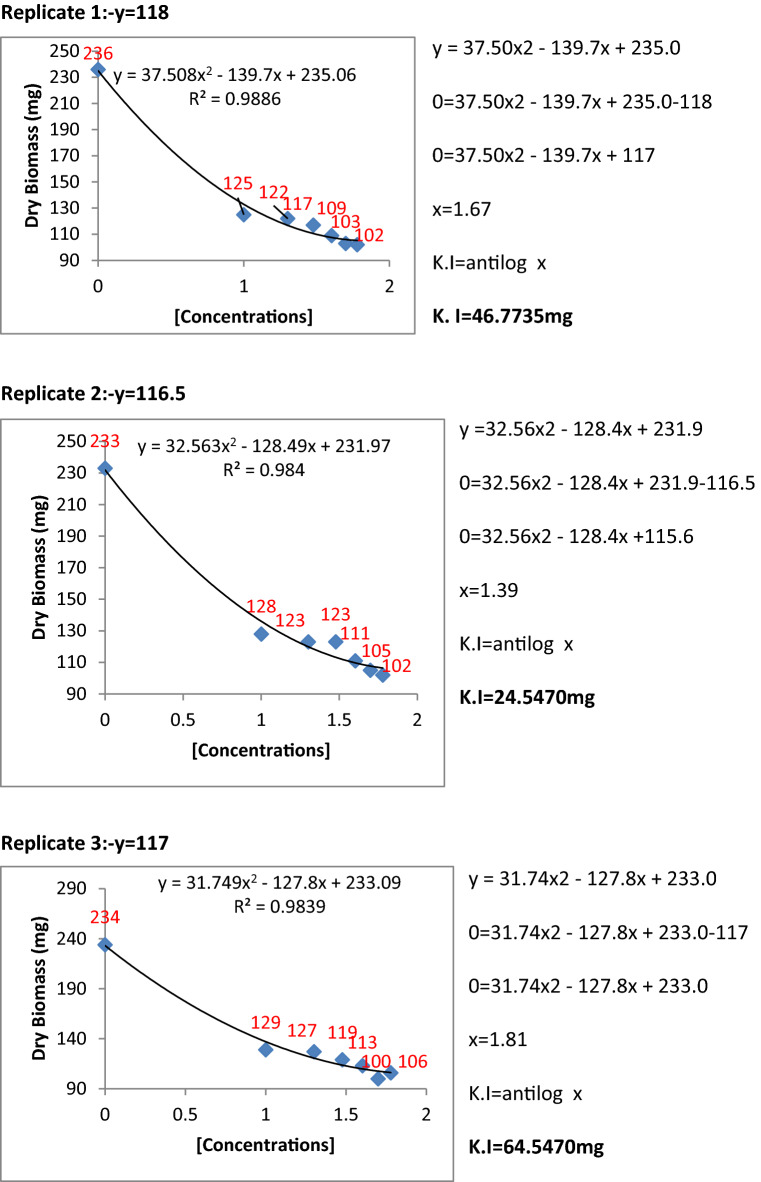
Kinetic constants of inhibition of *P. herbarum* by metabolites of *P. digitatum* (3 replicates).

**Figure 13:**
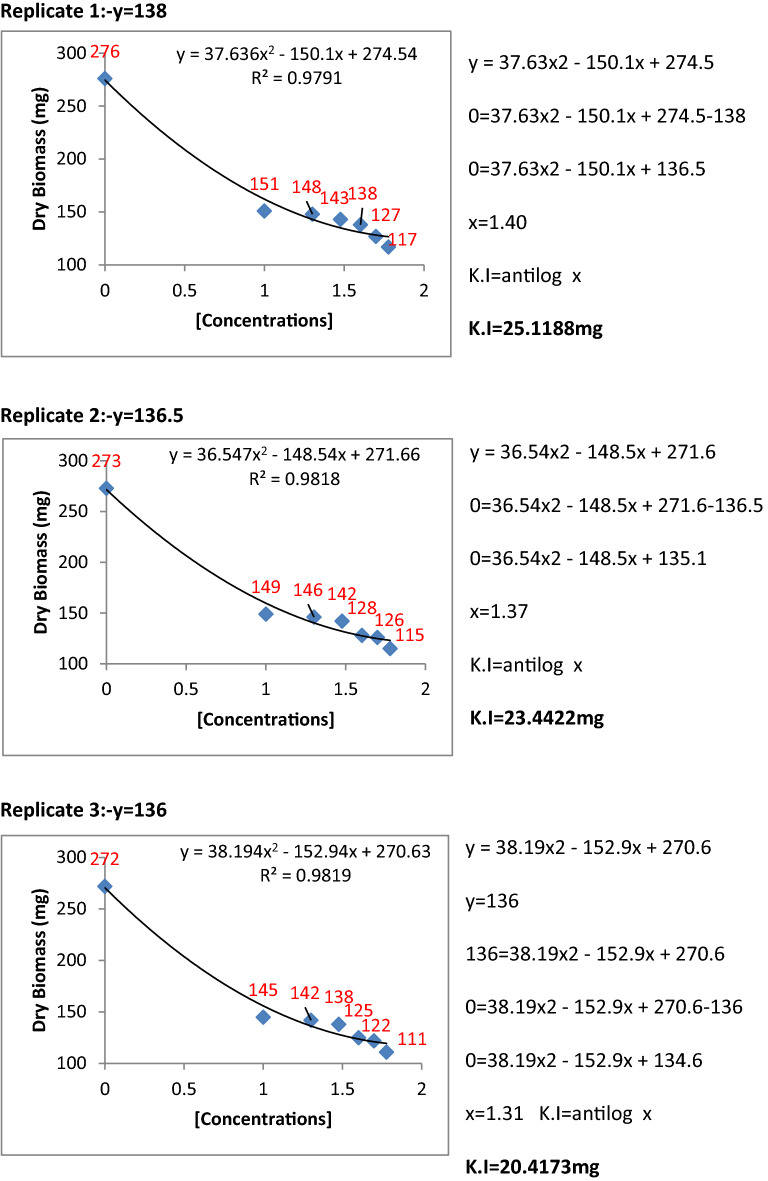
Kinetic constants of inhibition of *P. herbarum* by metabolites of *P. verrucosum* (3 replicates).

**Figure 14:**
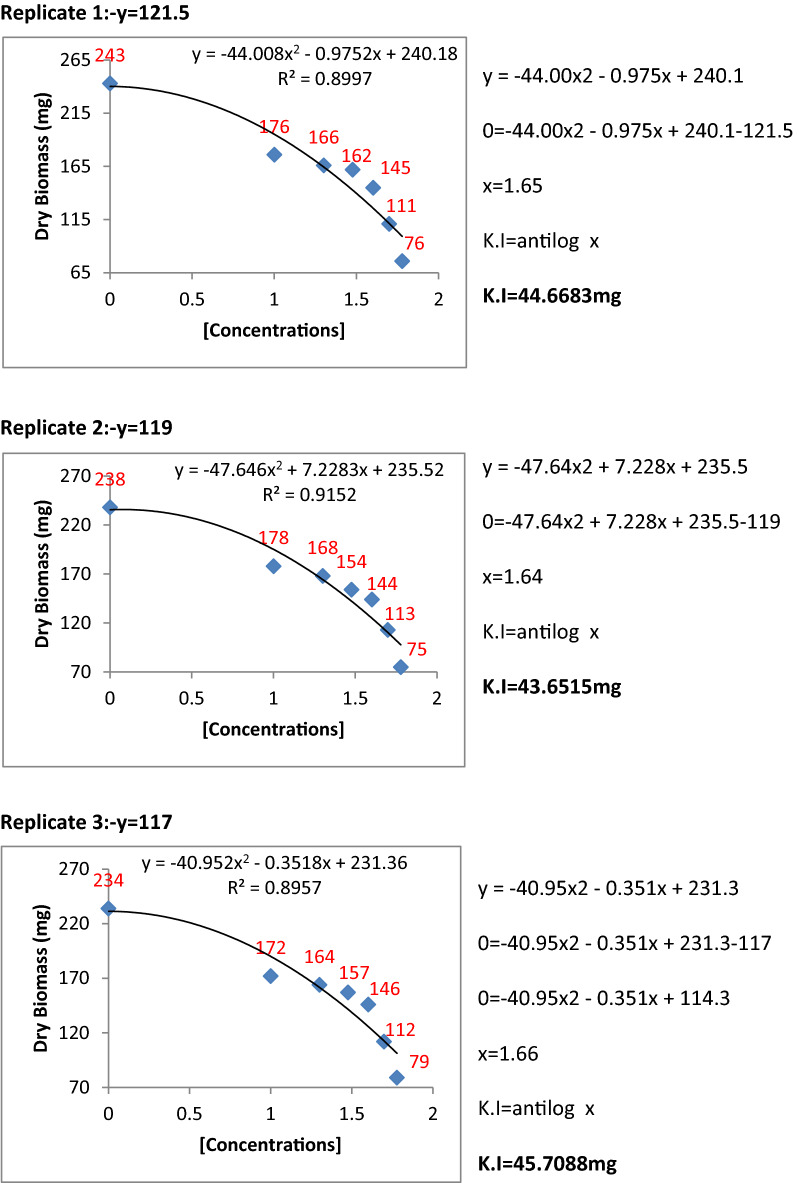
Kinetic constants of inhibition of *P. herbarum* by metabolites of *P. crustosum* (3 replicates).

**Figure 15:**
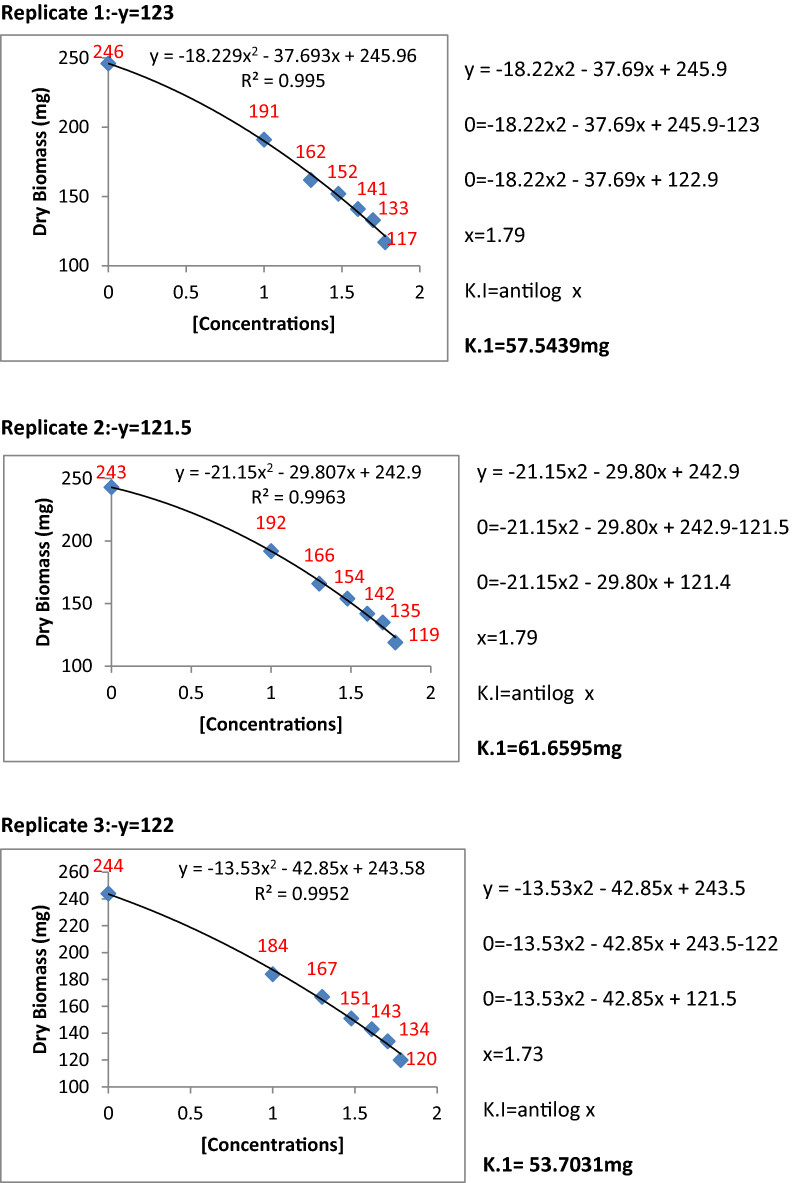
Kinetic constants of inhibition of *P. herbarum* by metabolites of *P. oxalicum* (3 replicates).

The metabolic extracts of Penicillium species were found to be very effective and highly significant against fungal pathogens. To find the inhibition constants regression equation was used and determined the regression of fungal biomass production versus various concentrations of metabolite extracts of Penicillium species. From the regression equation, the reduced fungal biomass by 50% of the control was determined by all the concentrations of metabolite extracts of Penicillium species (Figs. 11, 12, 13, 14 and 15). Calculated K.I values of the pathogen were presented in Table 1. The K.I results based upon all treatments provided a range of 22.54–57.64. The metabolite extracts of all species of *Penicillium (P. janczewskii, P. verrucosum, P. crustosum, P. digitatum*, and *P. oxalicum)* showed the K.I values 22.54, 23.56, 44.70, 45.64, 57.64, respectively. The metabolite extract concentration of *P janczewskii* depicted a minimum K.I value than the other 4 species which demonstrated that the fungal growth of *P. herbarum* was statistically most significantly inhibited by *P. janczewskii.*

**Table 1:** Kinetic constants for fungal biomass inhibition by *Penicillium* metabolites.

Pathogen	*Penicillium* species	K.I	SE
*Phoma herbarum*	*P. janczewskii*	22.54	± 3.856
	*P. digitatum*	45.64	± 11.865
	*P. verrucosum*	*23.56*	± 1.723
	*P. crustosum*	44.70	± 0.616
	*P. oxalicum*	57.64	± 2.30

### Effect of *Penicillium* metabolites on the expression of STE12 gene

**Figure 16:**
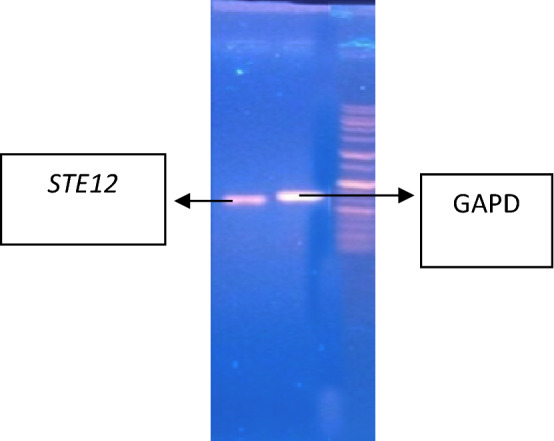
Agarose gel electrophoresis of GAPDH and STE12 genes amplified using DNA of *P. herbarum.*

The test pathogen (*P. herbarum*) has the ability to form appressorium. Thus, for the development of appressorium to penetrate into the host tissue *STE12* gene is required. *P. herbarum* was grown in malt extract employed with varied concentrations of metabolites of the most potent antagonist, *P. janczewskii* to evaluate the effect of Penicillium metabolites on the transcript level of *STE12* using Real-time reverse transcription PCR (qPCR). The *STE12* expression level was compared with the expression of the housekeeping gene, partial Glyceraldehyde-3-phosphate dehydrogenase (GAPDH) coding gene. In order to optimize the annealing temperature for selected *STE12* amplifying primers and housekeeping gene, a number of PCRs were conducted with a range of annealing temperatures from 50 to 65 °C using fungal genomic DNA as template. Optimum amplification of both pairs of genes was achieved at 60 °C (Fig. 16).

RNA of the pathogen grown in different concentrations of *P. janczewskii* metabolites was isolated using GeneAll® biotechnology kit and its concentration was determined. cDNA was synthesized from the extracted RNA from all the treatments of the pathogen and the concentration of cDNA was also measured using a NanoDrop® spectrophotometer.

#### Quantitative gene expression analysis by real-time PCR

qPCR results clearly demonstrated the expression of *STE12* as well as *GAPDH* in all treatments of *P. herbarum*. However, different levels of expression were observed for the *STE12* gene in different treatments. The threshold (C_T_) value remained similar in all treatments which is a clear indication that an equal amount of cDNA was used for each reaction mixture. ∆∆Cq was calculated to check the relative expression of *STE12* genes with the *GAPDH* gene that showed unchanged expression by metabolites. ∆∆Cq values were used to calculate the % Knockdown (KD) of gene expression of the quantification cycle that indicated the increase or decrease in gene expression. An increase in the % Knockdown value means decreased expression level.

The expression pattern of the *STE12* gene as determined by the % Knockdown value in *P. herbarum* grown in various concentrations of *Penicillium janczewskii* metabolites is recorded in Table 2. It was revealed from the results that there is a decrease in expression Knockdown values with the increase in metabolite concentrations. About 51.479% Knockdown of *STE12* encoding gene was noticed when the *P. herbarum* was grown in 10% metabolites stress. At 20% metabolites concentration, the % Knockdown value was decreased to 43.224% hence increase in expression of the *STE12* gene was recorded. The results exhibited a further decrease in % KD values 40.67, 38.01, 35.97, and 33.41 in *P. herbarum* employed with 30, 40, 50, and 60% metabolite concentrations, respectively.

**Table 2:** % Knockdown values of real-time PCR of *Phoma herbarum* treatments against *Penicillium janczewskii*.

Treatments	Cq	Cq	ΔCq	ΔCq Expression	Mean ΔCq	ΔΔCq	% KD
	GAPDH	STE12			Normalized		
P0	24.31	24.91	0.6	0.659754	0.802923		
	23.78	24	0.22	0.858565			
	22.97	23.1	0.13	0.913831			
P10	16.93	17.33	0.4	0.757858	0.389582	0.485205	51.47949
	16.18	18.01	1.83	0.281265			
	15.9	17.75	1.85	0.277392			
P20	12.27	13.32	1.05	0.482968	0.455861	0.567752	43.22478
	12	13.79	1.79	0.289172			
	11.77	12.33	0.56	0.678302			
P30	12.55	13.82	1.27	0.41466	0.476319	0.593231	40.67687
	12.98	14.01	1.03	0.48971			
	12.22	13.13	0.91	0.532185			
P40	13.67	14.97	1.3	0.406126	0.497695	0.619854	38.01462
	13.95	14.76	0.81	0.570382			
	13	13.91	0.91	0.532185			
P50	12.56	13.99	1.43	0.371131	0.514057	0.640232	35.9768
	13.67	14.34	0.67	0.628507			
	12.78	13.56	0.78	0.582367			
P60	20.22	21.34	1.12	0.460094	0.53465	0.66588	33.41204
	21	22.51	1.51	0.351111			
	21.01	21.09	0.08	0.946058			

**Figure 17:**
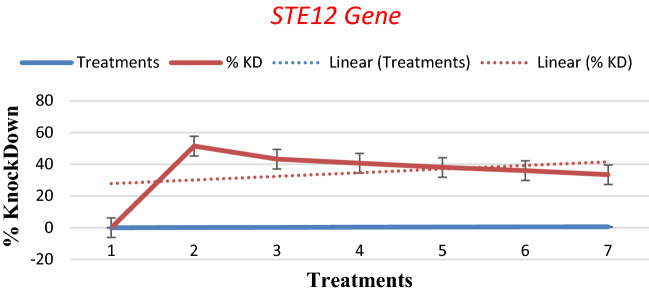
Gene expression of *STE12 Gene* in *P. herbarum* against *Penicillium janczewskii.*

Figure 17 also displayed a similar pattern of *STE12* gene expression. At 0% concentration, the gene was amplified and detected. It was observed that as the concentrations were increased from 10 to 60% (maximum), the % Knockdown values decreased accordingly.

#### In-silico tools predicted direct involvement of Ste12 in MAPK signaling pathway

**Figure 18:**
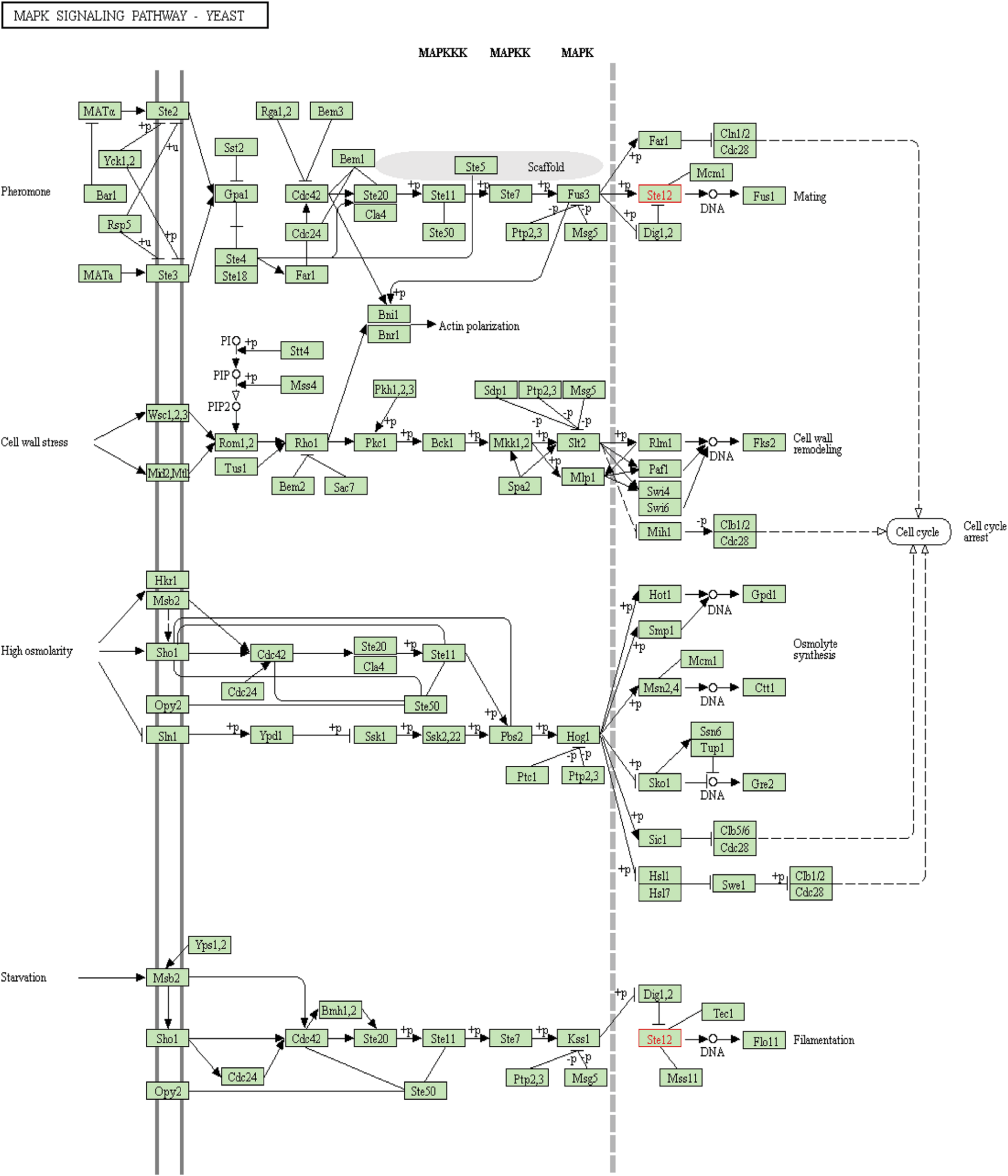
MAPK signaling Pathway in filamentous fungi developed by using KEGG pathway database^50^ with permissions from Kanehisa Laboratories (KEGG orthology entry: K11215): The cascade of MAPK is activated through kinases and phosphatases in all eukaryotes. In fungi, it maintains the survival rate during various stress conditions through adaptations. Ste12 a fungal transcription factor involves in response to stimulation through pheromones and low nutrient conditions to endure with increased mating and filamentation ability respectively. Source: https://www.genome.jp/dbget-bin/www_bget?sce:YHR084W.

The results depicted MAPK signaling pathway cascade prevalent in filamentous fungi that were predicted through the in-silico tool, KEGG database. It was revealed that the transcription factor Ste12 has direct involvement in this pathway in response to pheromones and under low nutrient conditions (starvation) as shown in Fig. 18.

## Discussion

Mung bean is a fast-growing legume and is a good source of dietary protein, calcium, and iron. The yield of this agricultural crop is most commonly reduced by plant diseases. Among these diseases, the harm caused by plant pathogens impacts about 13% of yield losses per annum worldwide^51^. Among the number of pathogens, over and above 80% of plant diseases and momentous damages to the human diet are due to Fungi. Among different constraints; the most distressing disease of mung bean is leaf spot disease which is caused by innumerable mycological pathogens including *Alternaria, Phoma, Drechslera,* etc. Different techniques are in use for the prevention and/or control of plant diseases^52^. The biological control method is considered to be the most effective way to regulate fungi*.* Many plant species and various plant extracts e.g., eucalyptus, neem, garlic, black pepper, ginger, and many weeds have been analyzed for their antifungal potential with the intent of ascertaining environmentally harmless and cost-effective alternatives for the control of diseases^32,33,53,54^. Besides the plant extracts; various microorganisms particularly a number of fungal and bacterial species are known to have biocontrol activity^55–57^. Most of the antagonistic species of fungi and bacteria are well known to have effective biocontrol potential against various plant diseases^33^, especially in fruits and crop plants. Presently, the antifungal potential of metabolites extracts of 5 *Penicillium* species was tested against *P. herbarum* to evaluate their biological control potential. It was obvious from the findings that *Penicillium* species had the innate capability to induce antagonistic effects on the fungal pathogen. The relative intensity of this effect however varied with the species involved, as well as the particular concentrations of the extract employed. The metabolites extract of all Penicillium species significantly reduced the fungal biomass of the target pathogen. Penicillium species are well recognized to secrete a wide range of bioactive metabolites including siderophore, indole acetic acid (IAA), hydrocyanic acid (HCN), lipase, protease, and β-1,3 glucanase that not only facilitate iron uptake in plants but also mediate disease suppression^58^. In accordance with our present study, Alam and coworkers^59^ scrutinized the effect of *Penicillium* sp. EU0013 on Fusarium wilt disease. In dual culture experiments, EU0013 significantly inhibited the growth of Fusarium wilt pathogens on tomato (*Solanum lycopersicum*L.) and cabbage (*Brassica oleracea*L.). In a parallel study, Sreevidya and Gopalakrishnan^45^ reported the production of citrinin, a secondary metabolite, by *Penicillium citrinum* VFI-51 which proved to be responsible for regulating the Botrytis gray mold disease in chickpea.

It was observed that the effectiveness of the extracts was found to be associated with the resistance or susceptibility offered by different species of *Penicillium.* Presently, the percentage inhibition in biomass production was different for all Penicillium species. Inhibition in biomass production indicated that antifungal compounds may be produced by Penicillium species. However, variation in inhibition in biomass production showed differences in the efficiency of each Penicillium species against the pathogen. In a contemporary study, Mamat et al.^60^ evaluated 7 strains of *Penicillium oxalicum* against *Colletotrichum gloeosporioides.* Their findings indicated that among the seven endophytic potential strains, *P. oxalicum* T3.3 demonstrated the most potent antagonistic activity towards *C. gloeosporioides* by producing the large inhibition zone against the pathogen tested. Several other workers also reported *P. oxalicum* to produce an inhibition zone against a wide range of pathogenic fungi during a dual culture test^58,61,62^.

Currently, it was evident from the antifungal bioassays that all the employed concentrations of metabolite extract suppressed the fungal growth but the highest concentration of metabolites of all Penicillium species suppressed biomass production up to 90 to 95%. In a similar kind of research Trichoderma species viz., *Trichoderma viride*, *Trichoderma aureoviride, Trichoderma reesei, Trichoderma koningii,* and *Trichoderma harzianum* showed a good potency as an antifungal agent against *Alternaria citri.* Among all culture filtrates *T. harzianum* was found to be highly effective in subduing the growth of test fungal species up to 93%^63^.

In the present study, although a significant reduction in biomass production of the pathogen was observed by all concentrations of all metabolites types, however, *P. janczewskii* proved the most toxic for the fungal growth as it induced more than 50% inhibition in fungal biomass production with the least KI values. These findings are in good agreement with the previously published results that showed the extracts of *Trichoderma* isolates had good activity against the plant pathogenic fungus *Alternaria alternata*^64^.

Real-time PCR is the most efficient molecular tool in determining the role of genes in disease development^65^. For real-time PCR, reference genes with stable expression under stimuli or stress play a vital role in comparison and conclusion. The most widely used reference genes are *β-tubulin*^66^*, GAPDH*^67^, and actin^65^. Presently, the *GAPDH* gene was used as a housekeeping gene and the narrow range of cycle threshold (Ct) was observed under all tested conditions. Results of the present study revealed that the higher the concentration of the metabolite more is the transcript for the *STE12* gene in *P. herbarum*. It has been reported that MAPK homologs also regulate the conidia formation and under stress, organisms try to form more spores which could be the possible reason for lower Knockdown values in high metabolites conditions^68^. In another study by Park and colleagues^65^, it was observed that under oxidative stress the expression of the *STE12* gene is unregulated in rice blast fungus *Magnaporthe oryzae.* The possibility of new breakthroughs in the control of pathogens involves a better understanding of the virulence mechanisms deployed by *E. rostratum* as pathogen aggressiveness is controlled by the interactions of several genes that react to signals that appear during host–pathogen interactions^24,65^.

The pathogenicity of the *P. herbarum* was carried out through the appressorium which is a specialized cell that has a high ability for an invasion via conidia or hyphae. In fungi, the presence of external stimuli including pheromones, starvation, hyper-osmolarity, and stress condition activates the Mitogen-activated protein kinase (MAPK) pathway for survival^69^. The MAPK pathway is a major signaling system that controls a variety of biological functions in fungi such as cell cycle, growth, differentiation of cells, virulence, and increase in their survival^70^. MKPs play a role in mycelial growth and pathogenicity in filamentous fungi, activated through many transcription factors^71^. Ste12 is a fungal transcription factor responsible for the regulation of other genes and also induces mycelial adaptations during infection as depicted in Fig. 13. In response to the pheromones, phosphorylated Fus3 (Mitogen-activated protein kinase FUS3) activates the transcription factor Ste12 to increase the interaction with DNA to transcribed Fus1 (Mitogen-activated protein kinase FUS1) which will adapt the fungi to enhance its mating ability^30^. While in the environment of deprived nutrients, MAPK-pathway permits adjustment by activating Ste12 through Kss1 (Mitogen-activated protein kinase gene Kss1) to increase the transcription of Flo11 which has a crucial role in filamentation to increase access to food^72^. Treatment with secondary metabolites of Penicillium as biocontrol reduced the expression of mRNA of Ste12 which will interrupt the MAPK cascade to attain adaptive responses during infection of *P. herbarum*.

Thus, the bioassays in the present study conclude the utility of *Penicillium* metabolites that possessed the strongest antifungal potential against *P. herbarum* as these metabolites are the precious benediction of nature for disease management against the most devastating pathogen by acting as defense materials against it.

## Materials and methods

*Phoma herbarum*, isolated and identified as a leaf spot pathogen of mungbean in Pakistan^73^ was obtained from the Fungal Biotechnology research lab, Department of Plant Pathology, Faculty of Agricultural Sciences, University of the Punjab, Lahore, Pakistan. The biological control potential of the metabolites of five Penicillium species was assessed to control *Phoma herbarum.*

### Selection of fungus as a biocontrol agent

Five Penicillium strains (*Penicillium oxalicum* FCBP-1075*, Penicillium crustosum* FCBP-1159*, Penicillium janczewskii* FCBP-1179*, Penicillium digitatum* FCBP-1160*,* and *Penicillium verrucosum* FCBP-1162 A-3) were acquired from the First Fungal Culture Bank of Pakistan, Department of Plant Pathology, Faculty of Agricultural Sciences, University of the Punjab, Lahore on freshly prepared MEA Petri plates. These cultures were utilized in subsequent metabolites extract preparation.

### *Penicillium* metabolites extraction

To prepare a stock solution of fungal metabolites extract, 2% malt extract was prepared and a disc of about 5 mm from freshly revived culture plates was inoculated in each flask containing 100 mL broth medium. The same method was repeated for other strains of Penicillium and left to grow at 25 ± 2 °C for about two weeks. After 15 days, with the completion of fungal mycelium, the metabolites were filtered through 2–threefold of sterilized Whatman filter paper 4 under aseptic conditions and preserved at 4 °C for subsequent use as a biocontrol agent^74^.

#### Fungal growth assay

To perform fungal growth assay in MEA broth, 7 concentrations viz., 0%, 10%, 20%, 30%, 40%, 50%, and 60% of each Penicillium strain were prepared in 60 mL of 2% broth medium containing 1.2gm malt extract per treatment flask. For fungal growth assays, these seven concentrations 0%, … 60% were prepared by adding 0, 6, 12, 18, 24, 30, 36 mL stock solutions (metabolite filtrate) to each flask containing 60 mL, 54 mL, 48 mL, 42 mL, 36 mL, 30 mL, 24 mL of broth, respectively and the final volume was raised up to 60 mL. 0% concentration was selected as the control treatment to compare the results of antifungal activity. The metabolite extract concentrations were divided to make three replicates of each concentration and subsequently simmered into an autoclave at 60 °C for about 20 min with zero pressure^74^. After simmering, 1 disc of 0.2 mm size from pure fungal culture plates of the pathogen was inoculated in every treatment separately and incubated at 25 ± 3 °C for 8–10 days until the growth in the control treatment reached its maximum. After 10 days, the fungal biomass was collected from all the replicates on the pre-weighed filter papers, oven-dried at 55–60 °C and dry biomass was obtained. The dry weight of biomass was used to assess the efficacy of each concentration of metabolites of all *Penicillium* strains. Percentage decreases or increases in fungal biomass due to various concentrations of employed extracts were determined by the following formula:

$$ {\text{Biomass inhibition }}\left( \% \right) \, = \,\frac{{{\text{Biomass in control }} - {\text{ Biomass in treatment}}}}{{\,\,{\text{Biomass in control}}}}\,\, \times \,\,100. $$

Percentage inhibition constants were evaluated by a decrease in fungal biomass with respect to various concentrations of metabolite extracts of different *Penicillium* strains using regression analysis.

### Effect of *Penicillium* metabolites on the expression of STE12 gene

The effect of different concentrations of selected *Penicillium* metabolite (with maximum antifungal potential) on the transcript level of StSTE12 was studied. The partial Glyceraldehyde-3-phosphate dehydrogenase (GAPDH) coding gene was selected as a housekeeping gene for comparison. The detail of the primers used in the study is presented in Table 3.

**Table 3:** Detail of genes and primers used for gene expression studies.

Sr. No	Gene	Primer name	Sequence (5′–3′)
1	*StSTE12*	StSTE12 (Forward)	5′- TCAACACGGTAGAGGAGAGCC-3′
		StSTE12 (Reverse)	5′- TCGTCACCCTCGAGATCTTCC -3′
2	*GAPDH*	GAPDH (Forward)	5′-CAA CGG CTT CGG TCG CAT TG-3′
		GAPDH (Reverse)	5′-GCC AAG CAG TTG GTT GTG C-3′

Primers were designed based on GenBank database information. Therefore, their specificity was checked by PCR amplification reactions using the fungal genomic DNA of the pathogen and the selected primer pair. The annealing temperatures of selected primers were also optimized.

#### Quantitative gene expression analysis

RNA from the fungus mycelia grown under selected treatments was isolated using a commercially available RNA isolation GeneAll® biotechnology kit and immediately treated with DNAase enzyme to avoid its degradation. To perform a Real-Time Polymerase Chain Reaction, isolated RNA was converted to complementary DNA (cDNA). The reaction was carried out at 55 °C for 1 h and stopped by incubating the mixture first at 85 °C for 5 min then on ice for 5 min. The synthesized cDNA was stored at − 20 °C until further used. Concentrations of the cDNA were measured using NanoDrop®. The cDNA was then diluted to make the concentration 200 ng/µl to ensure an equal amount of cDNA in the Real-Time PCR analysis. qPCR was conducted using two primers StSTE12 experimental primer and GAPDH as control primer because its expression remains the same throughout the experiment (Supplementary Fig. 1).

##### Real-time PCR

SYBR® Green Master Mix was used for quantitative gene expression studies in a 20 µL reaction mixture containing 2 µL of cDNA, 0.7 µL each of forward and reverse primer (10 µM), and 10 µL SYBR® green master mix. The PCR reaction was carried out as; one cycle at 95 °C for 10 min followed by 40 cycles each of denaturation at 95 °C for 15 s, annealing at 60 °C for 30 s, and elongation at 72 °C for 30 s.

#### In-silico studies

The Kyoto Encyclopedia of Genes and Genome (KEGG) database (https://www.genome.jp/kegg/) was used to predict the possible role of the Ste12 transcription factor in the MAPK signaling pathway. It’s an open-source database that includes 16 major databases providing a wide range of systematic genome information^50^.

## Supplementary Information

Supplementary Information.

## Data Availability

The data that support the findings of this study is contained within the manuscript.

## References

[1] Ali MZ, Khan MAA, Rahaman AKMM, Ahmed M, Ahsan AFMS (2010). Study on seed quality and performance of some mung bean varieties in Bangladesh. Int. J. Exp. Agric..

[2] Kumari R, Shekhawat KS, Gupta R, Khokhar MK (2012). Integrated management against root-rot of mung bean (*Vigna radiata* L.) Wilczek) incited by *Macrophomina*
*phaseolina*. J. Plant Path. Microbiol..

[3] Khan MA, Naveed K, Ali K, Ahmad B, Jan S (2012). Impact of mung bean-maize intercropping on growth and yield of mung bean. Pak. J. Weed Sci. Res..

[4] Scarmeas N, Stern Y, Tang MX, Mayeux R, Luchsinger JA (2006). Mediterranean diet and risk for Alzheimer’s disease. Ann. Neurol..

[5] Scarafoni A, Magni C, Duranti M (2007). Molecular nutraceutics as a mean to investigate the positive effects of legume seed proteins on human health. Trends Food Sci. Technol..

[6] Villegas R, Gao YT, Yang G, Li HL, Elasy TA, Zheng W, Shu XO (2008). Legume and soy food intake and the incidence of type 2 diabetes in the Shanghai Women’s Health Study. Am. J. Clin. Nutr..

[7] Khan AAI, Inam I, Ahmad F (2016). Yield and yield attributes of Mung bean (*Vigna*
*radiata* L.) cultivars as affected by phosphorous levels under different tillage systems. Cogn. Food Agric..

[8] Kudre TG, Benjakul S, Kishimura H (2013). Comparative study on chemical compositions and properties of protein isolates from mung bean, black bean and bambara groundnut. J. Sci. Food Agric..

[9] Chen MX, Zheng SX, Yang YN, Xu C, Liu JS, Yang WD, Chye ML, Li HY (2014). Strong seed-specific protein expression from the *Vigna radiata* storage protein 8SGα promoter in transgenic Arabidopsis seeds. J. Biotechnol..

[10] Tham DM, Gardner CD, Haskell WL (1998). Potential health benefits of dietary phytoestrogens: A review of the clinical, epidemiological, and mechanistic evidence. J. Clin. Endocrinol. Metab..

[11] Espín JC, García-Conesa MT, Tomás-Barberán FA (2007). Nutraceuticals: Facts and fiction. Phytochemistry.

[12] Fery RL (1990). The cowpea: Production, utilization, and research in the United States. Hort. Rev..

[13] Government of Pakistan. Agricultural statistics of Pakistan 2014–2015. Islamabad: Ministry of Food Security and Agricultural Research (2015).

[14] Pande, S. Integrated foliar diseases management of legumes 143–161 (2009).

[15] Chandrashekar, N., Guptha, O., Yelshetty, S., Sharma, O.P., Bhagat, S., Chattopadhay, C., Sehgal, M., Kumari, A., Amerasan, N., Sushil, S.N., Sinha, A.K., Asre, R., Kapoor, K.S. Sathyagopal, K. & Jeyakumar, P. Integrated pest management for chickpea. *Nat. Cen. Integ. Pest Manag. New Delhi, India*. 43 (2014).

[16] Satyagopal, K., Sushil, S.N., Jeyakumar, P., Shankar, G., Sharma, O.P., Boina, D.R., Sain, S.K., Lavanya, N., Sunanda, B.S., Asre, R., Kapoor, K.S., Arya, S., Kumar, S., Patni, C.S., Jacob, T.K., Santhosh, J., Eapen, C.N., Biju, K., Dhanapal, H., Ravindra, B.C., Hanumanthaswamy Raju, L.S., Babu, R., Sathyanarayana, L. & Latha, S. *AESA based IPM Package for Redgram.* National Institute of Plant Health Management, Rajendranagar, Hyderabad, India. 42 (2014a).

[17] Satyagopal, K., Sushil, S.N., Jeyakumar, P., Shankar, G., Sharma, O.P., Boina, D.R., Sain, S.K., Lavanya, N., Sunanda, B.S, Asre, R., Kapoor, K.S., Arya, S., Kumar, S., Patni, C.S., Jacob, T.K., Santhosh, J., Eapen, C.N., Biju, K., Dhanapal, H., Ravindra, B.C., Hanumanthaswamy Raju, L.S., Babu, R., Sathyanarayana, L. & Latha, S. *AESA based IPM Package for Blackgram and Greengram*. National Institute of Plant Health Management, Rajendranagar, Hyderabad, India. 43 (2014b).

[18] Armentrout VN, Downer AJ (1986). Infection cushion development by *Rhizoctonia solani* on cotton. Phytopathology.

[19] Dean RA, Talbot NJ, Ebbole DJ, Farman ML, Mitchell TK, Orbach MJ, Thon M, Kulkarni R, Xu JR, Pan H (2005). The genome sequence of the rice blast fungus *Magnaporthe grisea*. Nature.

[20] Dean RA (1997). Signal pathways and appressorium morphogenesis. Ann. Rev. Phytopathol..

[21] Xu JR, Hamer JE (1996). MAP kinase and cAMP signaling regulate infection structure formation and pathogenic growth in the rice blast fungus *Magnaporthe grisea*. Gen. Dev..

[22] Ruiz-Roldan MC, Maier FJ, Schäfer W (2001). PTK1, a mitogen-activated-protein kinase gene, is required for conidiation, appressorium formation, and pathogenicity of *Pyrenophora teres* on barley. Mol. Plant-Microbe. Interact..

[23] Takano Y, Kikuchi T, Kubo Y, Hamer JE, Mise K, Furusawa I (2000). The *Colletotrichum lagenarium* MAP kinase gene CMK1 regulates diverse aspects of fungal pathogenesis. Mol. Plant-Microbe Interact..

[24] Schamber A, Leroch M, Diwo J, Mendgen K, Hahn M (2010). The role of mitogen-activated protein (MAP) kinase signaling components and the Ste12 transcription factor in germination and pathogenicity of *Botrytis cinerea*. Mol. Plant Pathol..

[25] Gu Q, Zhang C, Liu X, Ma Z (2015). A transcription factor FgSte12 is required for pathogenicity in *Fusarium graminearum*. Mol. Plant Pathol..

[26] Zhao X, Mehrabi R, Xu JR (2007). Mitogen-activated protein kinase pathways and fungal pathogenesis. Eukaryot. Cell.

[27] Ebbole DJ (2007). *Magnaporthe* as a model for understanding host-pathogen interactions. Ann. Rev. Phytopathol..

[28] Rispail N, Di Pietro A (2010). The homeodomain transcription factor Ste12: Connecting fungal MAPK signaling to plant pathogenicity. Commun. Integr. Biol..

[29] Chou S, Lane S, Liu H (2006). Regulation of mating and filamentation genes by two distinct Ste12 complexes in *Saccharomyces cerevisiae*. Mol. Cell. Biol..

[30] Hoi JWS, Dumas B (2010). Ste12 and Ste12-like proteins, fungal transcription factors regulating development and pathogenicity. Eukaryot. Cell.

[31] Shabana P, Kumar VR (2000). Effect of extracts of some medicinal plants on the growth of *Alternaria triticina*. J. Phytolog. Res..

[32] Javaid AR, Shafique S, Shafique S, Riaz T (2008). Effect of rice extracts and residue incorporation on *Parthenium hysterophorus* management. Allelopath. J..

[33] Shafique S, Asif M, Shafique S (2015). Management of *Fusarium oxysporum* f. sp. capsici by leaf extract of *Eucalyptus citriodora*. Pak. J. Bot..

[34] Bajwa R, Shafique S, Anjum T, Shafique S (2004). Antifungal activity of allelopathic plant extracts IV: Growth response of *Alternaria alternata*, *Fusarium monilifrome* and *Drechslera hawaiiensis* to aqueous extract of *Parthenium hysterophorus* L. Int. J. Agric. Biol..

[35] Lorito, M., Woo, S., Iaccarino, M., Iaccarino, M. Ed., Idelson-Gnocchi, S.R.L. & Scala, F. *In Microrganisms Antagonists* **1,** 146–175 (2006).

[36] Woo SL, Scala F, Ruocco M, Lorito M (2006). The molecular biology of the interactions between *Trichoderma* spp., phytopathogenic fungi, and plants. Phytopathology.

[37] Olabiyi TI (2009). Diseases of Food Crops and their Control Principles.

[38] Martínez JL (2012). Natural antibiotic resistance and contamination by antibiotic resistance determinants: The two ages in the evolution of resistance to antimicrobials. Front. Microbiol..

[39] Shields JK, Atwell EA (1963). Effect of a mold, *Trichoderma viride*, on decay by four storage fungi. J. Nat. Prod..

[40] Santamarina MP, Jiménez M, Sanchis V, García F, Hernández E (1987). A strain of *Penicillium funiculosum* Thom with activity against *panonychus ulmi koch* (Acar.Tetranychida). J. Appl. Entomol..

[41] Jyoti, S. & Singh, D. P. Fungi as biocontrol agents in sustainable agriculture. In *Microorganisms and Environmental Management.* 171–195 (2016).

[42] Frisvad JC, Samson RA (2004). Polyphasic taxonomy of *Penicillium* subgenus *Penicillium*: A guide to the identification of food and air-borne terverticillate *Penicillia* and their mycotoxins. Stud. Mycol..

[43] Lucas EMF, Castro MCM, Takahashi JA (2007). Antimicrobial properties of sclerotiorin, isochromophilone VI and pencolide, metabolites from a Brazilian cerrado isolate of *Penicillium sclerotiorum* van Beyma. Braz. J. Microbiol..

[44] Zia Ullah S, Syed A, Abbas A, Amir M, Qureshi S (2015). Evaluation of *Penicillium* sp. Eu0013 for management of root rot disease of okra. Int. J. Biosci..

[45] Sreevidya M, Gopalakrishnan S (2016). *Penicillium citrinum* VFI-51 as a biocontrol agent to control charcoal rot of sorghum (*Sorghum bicolor* (L.) Moench). Afr. J. Microbiol. Res..

[46] El-Fawy MM, El-Sharkawy RMI, Abo-Elyousr KAM (2018). Evaluation of certain *Penicillium frequentans* isolates against Cercospora leaf spot disease of sugar beet. Egy. J. Biol. Pest. Cont..

[47] Liang LJ, Jeewon R, Dhandevi P, Durairajan SSK, Li H, Lin FC, Wang HK (2021). A novel species of *Penicillium* with inhibitory effects against *Pyricularia oryzae* and fungal pathogens inducing citrus diseases. Front. Cell. Infect. Microbiol..

[48] Hyder S, Gondal AS, Rizvi ZF, Iqbal R, Hannan A, Sahi ST (2022). Antagonism of selected fungal species against *Macrophomina **p**haseolina* (Tassi) goid, causing charcoal rot of Mungbean. Pak. J. Bot..

[49] Hossain MM, Sultana F, Kubota M, Koyama H, Hyakumachi M (2007). The plant growth-promoting fungus *Penicilliumsim plicissimum* GP17-2 induces resistance in *Arabidopsis thaliana* by activation of multiple defense signals. Plant Cell Physiol..

[50] Kanehisa M, Goto S (2000). KEGG: Kyoto encyclopedia of genes and genomes. Nucleic Acids Res..

[51] Fletcher J, Bender C, Budowle B, Cobb WT, Gold SE, Ishimaru CA, Luster D, Melcher U, Murch R, Scherm H, Seem RC, Sherwood JL, Sobral BW, Tolin SA (2006). Plant-pathogen forensics: Capabilities, needs, and recommendations. Microbiol. Mol. Biol. Rev..

[52] Chandler D, Bailey AS, Tatchell GM, Davidson G, Greaves J, Grant WP (2011). The development, regulation, and use of biopesticides for integrated pest management. Philos. Trans. R. Soc. B Biol. Sci..

[53] Bajwa R, Iftikhar S (2005). Antifungal activity of allelopathic plant extracts V1: In vitro control of fungal pathogens by aqueous leaf extracts of eucalyptus. Mycopath.

[54] Shafique S, Bajwa R, Shafique S, Akhtar N, Hanif S (2011). Fungitoxic activity of aqueous and organic solvent extracts of *Tagetes erectus* on phytopathogenic fungus *Ascochyta Rabiei*. Pak. J. Bot..

[55] Harman GE (2000). Myths and Dogmas of biocontrol changes in perceptions derived from research on *Trichoderma harzianum* T-22. Plant Dis..

[56] Harman GE, Howell CR, Viterbo A, Chet I, Lorito M (2004). *Trichoderma* species—Opportunistic avirulent plant symbionts. Nat. Rev..

[57] Baniasadi F, Bonjar GHS, Baghizadeh A, Karimi Nik A, Jorjandi M, Aghighi S, Farokhi PR (2009). Biological control of *Sclerotinia sclerotiorum*, the causal agent of sunflower head and stem rot disease, by use of soil-borne actinomycetes isolates. Am. J. Agric. Biol. Sci..

[58] Yang L, Xie J, Jiang D, Fu Y, Li G (2008). Antifungal substances produced by *Penicillium oxalicum* strain PY-1—potential antibiotics against plant pathogenic fungi. World J. Microbiol. Biotechnol..

[59] Alam SS, Sakamoto K, Inubushi K (2011). Biocontrol efficiency of *Fusarium* wilt diseases by a root-colonizing fungus *Penicillium* sp. Soil Sci. Plant Nutr..

[60] Mamat S, Shah UKM, Remli NAM, Shaari K, Laboh R, Rahman NAA (2018). Characterization of antifungal activity of endophytic *Penicillium oxalicum* T 3.3 for anthracnose biocontrol in dragon fruit (H*ylocereuss*p). Int. J. Environ. Agric. Res..

[61] Larena I, Sabuquillo P, Melgarejo P, De Cal A (2003). Biocontrol of *Fusarium* and *Verticillium* wilt of tomato by *Penicillium oxalicum* under greenhouse and field conditions. J. Phytopathol..

[62] Paul NC, Deng JX, Sang HK, Choi YP, Yu SH (2012). Distribution and antifungal activity of endophytic fungi in different growth stages of chili pepper (*Capsicum*
*annuum* L.) in Korea. J. Plant Pathol..

[63] Murtaza A, Shafique S, Anjum T, Shafique S (2012). In vitro control of *Alternaria citri* using antifungal potentials of *Trichoderma* species. Afr. J. Biotechnol..

[64] Javed A (2017). Management of Syzygium cumini Leaf Necrosis by Secondary Metabolites of Trichoderma.

[65] Park SY, Choi J, Lim SE, Lee GW, Park J, Kim Y, Lee YH (2014). Global expression profiling of transcription factor genes provides new insights into pathogenicity and stress responses in the rice blast fungus. A Peer-Rev. Open-Acc. J..

[66] Goh J, Kim KS, Park J, Jeon J, Park SY, Lee YH (2011). The cell cycle gene MoCDC15 regulates hyphal growth, asexual development and plant infection in the rice blast pathogen *Magnaporthe oryzae*. Fungal Gen. Biol..

[67] Mathioni SM, Beló A, Rizzo CJ, Dean RA, Donofrio NM (2011). Transcriptome profiling of the rice blast fungus during invasive plant infection and in vitro stresses. BMC Gen..

[68] Zhang SR, Hao ZM, Wang LH, Shen S, Cao ZY, Xin YY, Hou ML, Gu SQ, Han JM, Dong JG (2012). StRas2 regulates morphogenesis, conidiation and appressorium development in *Setosphaeria turcica*. Microbiol. Res..

[69] Sun Y, Liu WZ, Liu T, Feng X, Yang N, Zhou HF (2015). Signaling pathway of MAPK/ERK in cell proliferation, differentiation, migration, senescence, and apoptosis. J. Recept. Signal Transduct..

[70] Cargnello M, Roux PPJM, reviews, M.B. (2011). Activation and function of the MAPKs and their substrates, the MAPK-activated protein kinases. Microbiol. Mol. Biol. Rev..

[71] González-Rubio G, Fernández-Acero T, Martín H, Molina MJIJOMS (2019). Mitogen-activated protein kinase phosphatases (MKPs) in fungal signaling: Conservation, function, and regulation. Int. J. Mol. Sci..

[72] Chow J, Starr I, Jamalzadeh S, Muniz O, Kumar A, Gokcumen O, Cullen PJJG (2019). Filamentation regulatory pathways control adhesion-dependent surface responses in yeast. Genetics.

[73] Shafique S, Attia U, Akhtar N, Shafique S (2022). Isolation and Identification of a novel leaf necrotic pathogen of *Vigna radiata* in Pakistan. Pak. J. Bot..

[74] Shafique S, Shafique S, Javed A, Akhtar N, Bibi S (2019). Analysis of antagonistic potential of secondary metabolites and organic fractions of *Trichoderma* species against *Alternaria alternata*. Biocont. Sci..

