# Supplementary figures and images for: Management of mung bean leaf spot disease caused by Phoma herbarum through Penicillium janczewskii metabolites mediated by MAPK signaling cascade
Source: Sci Rep. 2023 Mar 3;13:3606. doi: 10.1038/s41598-023-30709-6 (PMC9984459; doi:10.1038/s41598-023-30709-6)

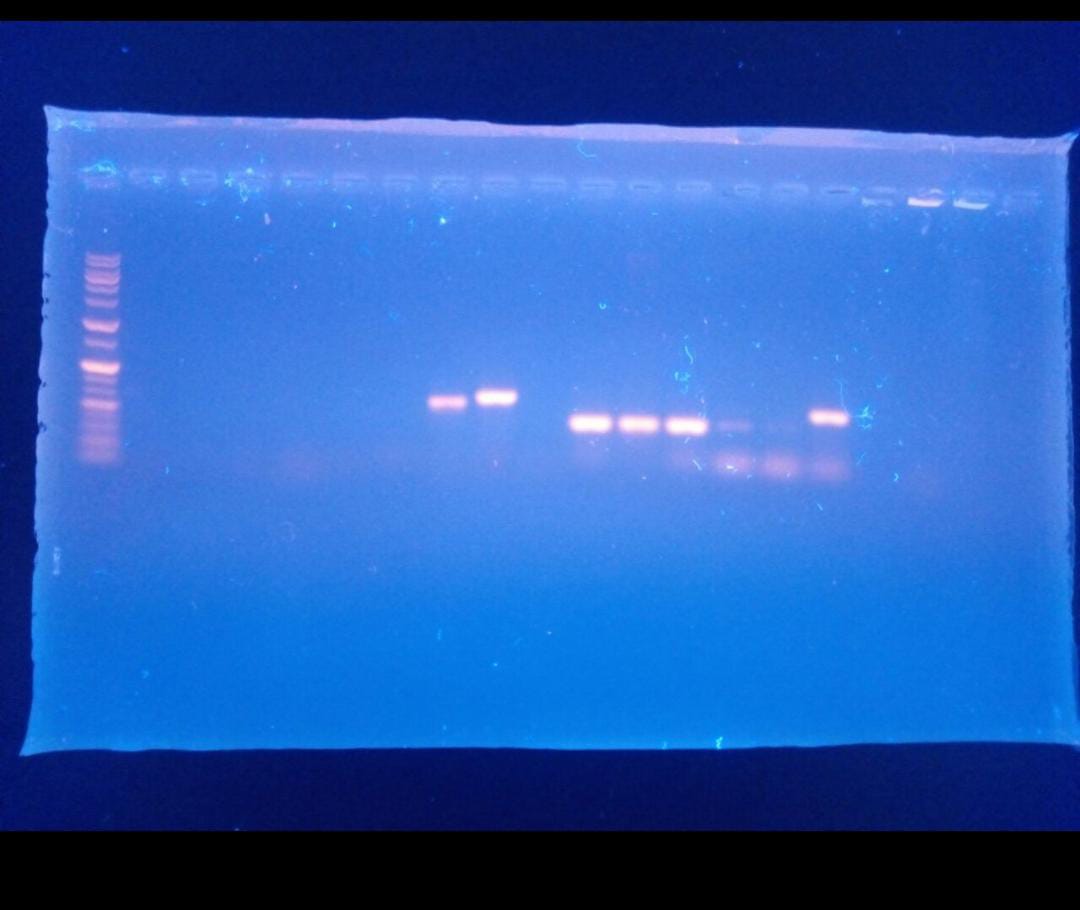

Supplement: Supplementary file 1 — Supplementary Information. [file 41598_2023_30709_MOESM1_ESM.jpg]
